# A comparative review on the well-studied GAT1 and the understudied BGT-1 in the brain

**DOI:** 10.3389/fphys.2023.1145973

**Published:** 2023-04-13

**Authors:** Manan Bhatt, Laure Gauthier-Manuel, Erika Lazzarin, Rocco Zerlotti, Christine Ziegler, Andre Bazzone, Thomas Stockner, Elena Bossi

**Affiliations:** ^1^ Department of Biotechnology and Life Sciences, University of Insubria, Varese, Italy; ^2^ Centre for Neuroscience—University of Insubria, Varese, Italy; ^3^ Department of Biophysics II/Structural Biology, University of Regensburg, Regensburg, Germany; ^4^ Center for Physiology and Pharmacology, Institute of Pharmacology, Medical University of Vienna, Waehringerstr, Vienna; ^5^ Nanion Technologies GmbH, Munich, Germany

**Keywords:** GABA, transporter, SLC6 neurotransmitter transporters, GAT1, BGT-1, structure-function

## Abstract

γ-aminobutyric acid (GABA) is the primary inhibitory neurotransmitter in the central nervous system (CNS). Its homeostasis is maintained by neuronal and glial GABA transporters (GATs). The four GATs identified in humans are GAT1 (SLC6A1), GAT2 (SLC6A13), GAT3 (SLC6A11), and betaine/GABA transporter-1 BGT-1 (SLC6A12) which are all members of the solute carrier 6 (SLC6) family of sodium-dependent transporters. While GAT1 has been investigated extensively, the other GABA transporters are less studied and their role in CNS is not clearly defined. Altered GABAergic neurotransmission is involved in different diseases, but the importance of the different transporters remained understudied and limits drug targeting. In this review, the well-studied GABA transporter GAT1 is compared with the less-studied BGT-1 with the aim to leverage the knowledge on GAT1 to shed new light on the open questions concerning BGT-1. The most recent knowledge on transporter structure, functions, expression, and localization is discussed along with their specific role as drug targets for neurological and neurodegenerative disorders. We review and discuss data on the binding sites for Na^+^, Cl^−^, substrates, and inhibitors by building on the recent cryo-EM structure of GAT1 to highlight specific molecular determinants of transporter functions. The role of the two proteins in GABA homeostasis is investigated by looking at the transport coupling mechanism, as well as structural and kinetic transport models. Furthermore, we review information on selective inhibitors together with the pharmacophore hypothesis of transporter substrates.

## 1 Introduction

The primary inhibitory neurotransmitter in the mammalian central nervous system (CNS) is γ-aminobutyric acid (GABA). Its presence in the brain was discovered independently by three groups in the year 1950 (Awapara et al., 1950; Roberts and Frankel, 1950; Udenfriend, 1950). GABA is estimated to be the neurotransmitter in 60%–75% of the synapses in the CNS (Fagg and Foster, 1983), and in about 30% of synapses in the brain (Durkin et al., 1995). In the cerebral cortex, GABA is involved in the control of the balance between excitation and inhibition, and consequently in network synchronization, information processing, and neuronal plasticity and excitability (Krnjević and Schwartz, 1967; Blatow et al., 2003; Conti et al., 2004; Imbrosci and Mittmann, 2011; Brady et al., 2018; Bi et al., 2020; Xu et al., 2020). Alteration of GABA homeostasis plays a significant role in several neuropathological diseases including epileptic seizures, Alzheimer’s, Parkinson’s, Huntington’s disease, and multiple sclerosis (Wojtowicz et al., 2013; Cawley et al., 2015; Rozycka and Liguz-Lecznar, 2017; Zafar and Jabeen, 2018).

The four human GABA transporters GAT1 (SLC6A1), BGT-1 (SLC6A12), GAT2 (SLC6A13), and GAT3 (SLC6A11) are part of the solute carrier 6 (SLC6) family. The GATs are expressed in both presynaptic neurons and surrounding glial cells: GAT1 is the most abundantly expressed GABA transporter, while the expression of GAT2 is the lowest in the CNS (Conti et al., 1999). To terminate GABAergic signaling GABA is either transported back into the releasing neuron (80%) or into the surrounding glial cells (20%), where it is metabolized further into glutamine (Zafar and Jabeen, 2018).

The selective inhibition of GABA uptake into astrocytes would lead to an increase in neuronal levels by elevated neuronal uptake. This indirect potentiation of neuronal GABA uptake has therapeutic importance as it ameliorates conditions that are caused by low GABA concentration in the GABAergic synapse (Schousboe et al., 2014; Patel et al., 2015). Multiple selective GABA inhibitors have been developed and are used as therapeutics (Kickinger et al., 2021). Among GATs, GAT1 has been investigated extensively, while BGT-1 is understudied and requires well-directional additional research to obtain a fair understanding of its structure, function, and role in human physiology. Using the research model of GAT1, several questions for BGT-1 could be inferred or answered. Here, we present a comprehensive review that focuses on GAT1 and BGT-1 with the aim to help the community inspire research in BGT-1 by inference from GAT1 data. We cover recent knowledge of GAT1 and BGT-1 on brain distribution, structure-function, and their roles in treating neurological and neurodegenerative disorders. We focus on the binding sites for Na^+^, Cl^−^, and substrates, and highlight specific molecular determinants of transporter functions and consequences for GABA homeostasis. We discuss the transport coupling mechanism, computational structural modelling, the recent cryo-EM structure (Motiwala et al., 2022; Kanner and Dayan-Alon, 2023), and define the structure of the binding site for GAT1 (Joseph et al., 2022). We also summarize information on GAT1 and BGT-1 specific inhibitors as well as the pharmacophore hypothesis of transporter substrates.

## 2 Historical perspective of GABA transporters

The first GAT was purified from rat brain in 1986 and cloned in 1990 (Radian et al., 1986; Guastella et al., 1990). It consists of 599 amino acids, twelve transmembrane helices, and has an apparent K_0.5_ in the μM range. The human GAT1 was also cloned in the same year (Nelson et al., 1990). Thereafter GAT2 and GAT3 were cloned from rat and human brain (Borden et al., 1992; Lopez-Corcuera et al., 1992; Borden et al., 1994). And lastly, the fourth GABA transporter was cloned from Madin-Darby canine kidney (MDCK) cells. This transporter is regulated by hypertonicity and was shown to transport the osmolyte betaine in addition to GABA, thus was called betaine/GABA transporter 1 (BGT-1) (Yamauchi et al., 1992). The human orthologue of BGT-1 was also cloned 2 years later from brain and kidney (Borden et al., 1994; Rasola et al., 1995). Like other SLC6 transporters, the transport function of GATs is energized by the transmembrane gradient of Na^+^ (Guastella et al., 1990).

The four GABA transporters were also identified and cloned in mice and named sequentially as GAT1, GAT2, GAT3, and GAT4 (Liu et al., 1992; Liu et al., 1993). This differentiation of nomenclature (See Table 1) can be confusing and was only resolved by the introduction to the systematic nomenclature of the SLC6 transporter family (Hediger et al., 2004). To avoid any misinterpretation caused by this ambiguity, in this work we will use nomenclature as per the human paralogues.

**TABLE 1:** Nomenclature, uptake substrates, inhibitors, and tissue expression of GABA transporters.

SLC6 gene	Species	Currently accepted name	Known uptake substrates	Known uptake inhibitors	Tissue expression	References
SLC6a1	Human	GAT1	GABA, nipecotic acid, guvacine, ACHC	Nipecotic acid, L-DABA, guvacine, SKF-89976a, tiagabine, ACHC, THPO, Cl-966, NO-711, EF1502, LU-32-176B, EGYT-3886	Cerebellar cortex, medulla, hippocampus, thalamus, hypothalamus, olfactory bulb, basal ganglia, cerebellum, interpeduncular nucleus, substantia nigra, basal forebrain, retina.	Augood et al. (1995), Yasumi et al. (1997), Eulenburg and Gomeza (2010), Jin et al. (2011), Ghirardini et al. (2018), Fattorini et al. (2020b)
	Rat	GAT1				
	Mouse	GAT1				
SLC6a12	Human	BGT-1	GABA, betaine	EGYT-3886, EF1502, bicyclo-GABA, NNC 05-2045, NNC05-2090	Liver, kidney, cerebral cortex, cerebellum, brainstem, hippocampus, leptomeninges.	Lopez-Corcuera et al. (1992), Yamauchi et al. (1992), Borden, 1996b, Jin et al. (2011), Zhou and Danbolt (2013), Kempson et al. (2014), Lie et al. (2020)
	Rat	BGT-1				
	Mouse	GAT2				
SLC6a13	Human	GAT2	GABA, nipecotic acid, ß-alanine	Nipecotic acid, L-DABA, guvacine, EGYT-3886	Liver, kidney, leptomeninges, pia mater, arachnoid complex.	Zhou et al. (2012), Zhou and Danbolt (2013)
	Rat	GAT2				
	Mouse	GAT3				
SLC6a11	Human	GAT3	GABA, nipecotic acid, ß-alanine	Nipecotic acid, L-DABA, guvacine, EGYT-3886, SNAP-5114, NNC-05-2045	Brainstem, cerebellum, cortex frontalis, cortex occipitalis, hippocampus, olfactory bulb, retina, hypothalamus, midline thalamus, medulla oblongata, basal forebrain, striatum.	Durkin et al. (1995), Jin et al. (2011), Ghirardini et al. (2018)
	Rat	GAT3				
	Mouse	GAT4				

## 3 Expression and localization

**FIGURE 1:**
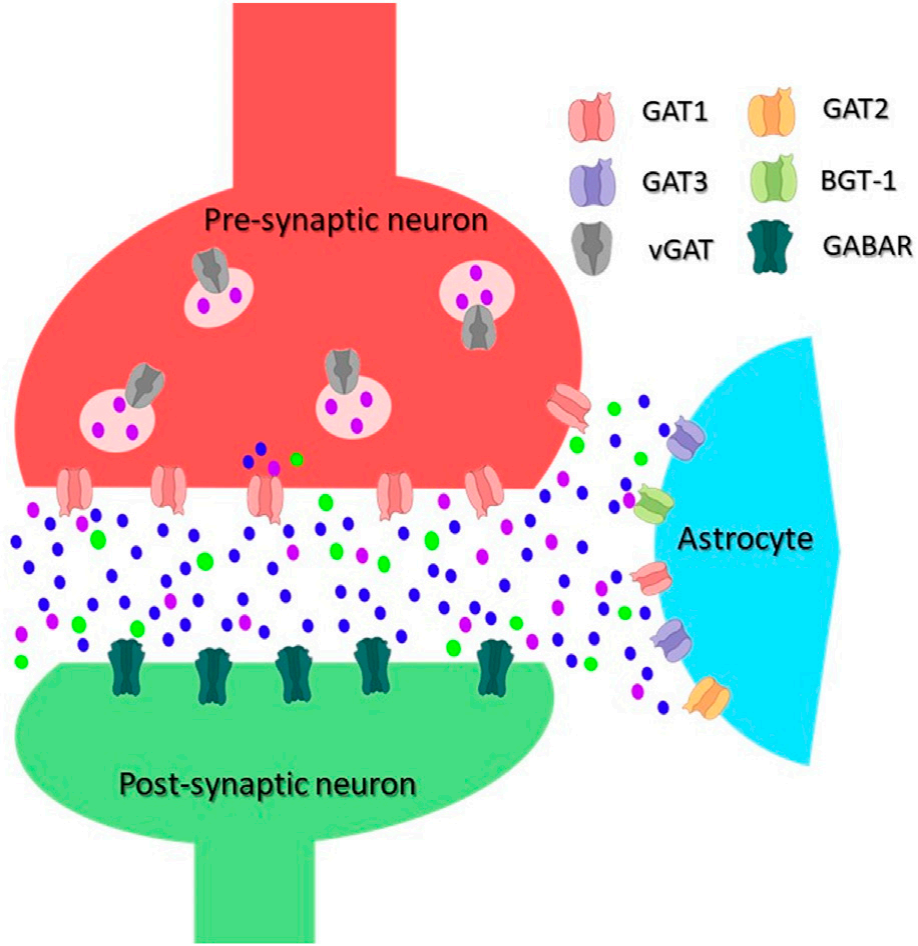
Scheme of an inhibitory synapse showing the distribution of GATs. In the pre-synaptic terminal of GABAergic neurons, GABA is synthesized from glutamate and packaged into vesicles by vesicular GAT (vGAT). GABA is released into the synaptic cleft upon depolarization of the plasma membrane. An inhibitory signal can be generated in the post-synaptic neuron by its binding to and opening of the ionotropic GABA receptors (GABAR). Released GABA is removed from the synaptic cleft by GATs, the transport is energized by co-transport of Na^+^/Cl^−^. The main GABA transporter in neurons is GAT1, whereas in astrocytes, mainly GAT3 but also GAT2 and BGT-1 are responsible for the GABA transport. GABA molecules are shown as purple dot, Na^+^ ions as blue dot, and Cl^−^ as green dot.

GATs are localized both in astrocytes and neurons (See Figure 1); in which they are present at the synaptic level and/or extra synaptically. Their tissue expression is reported in Table 1. GAT1 shows the highest expression rate among all GATs in the CNS, predominantly in the adult frontal cortex (Conti et al., 2004). GAT1 is expressed in GABAergic axon terminals, astrocytes, oligodendrocytes, and microglia (Minelli et al., 1995; Melone et al., 2015; Fattorini et al., 2020a). Expression of GATs changes with life (Sundman-Eriksson and Allard, 2006; Banuelos et al., 2014; Pandya et al., 2019; Khozhai, 2020). In the adult CNS the main localization of GAT-1 is neuronal and of GAT3 on the glial cells, whereas in the neonatal mammalian brain a larger abundance of GAT-1 was reported in astrocytes and of GAT3 in neurons. The early expression of GATs is most likely related to the high cytoplasmic Cl^−^ concentration at the initial brain developmental stages and the unusual roles of GABA in some brain regions, where it occasionally excites immature neurons. The function of GABA becomes inhibitory with the increased expression of the potassium-chloride exporter KCC2 (Borden, 1996a; Yan et al., 1997; Rivera et al., 1999; Minelli et al., 2003; Conti et al., 2004; Ben-Ari et al., 2012; Kilb, 2012). The distribution of BGT-1 in the human tissues differs from the expression pattern of the other GABA transporters, which is linked to its unique ability to transport the osmolyte betaine. The localization of BGT-1 in the brain is not well defined. Based on data from cultured astrocytes and astrocytoma lines, the expression of BGT-1 was reported at the blood-brain barrier and brain endothelium (Takenaka et al., 1995; Bitoun and Tappaz, 2000; Ruiz-Tachiquin et al., 2002). Interestingly, BGT-1 was also found in dendritic spines of glutamatergic synapses, showing a possible localization at extra-synaptic regions (Zhu and Ong, 2004a; Zhu and Ong, 2004b). The importance of BGT-1 for regulating brain GABA levels might be limited, because of the low BGT-1 expression, the slow turnover rates, and the low affinity for GABA in comparison to GAT1. Hence, suggesting that BGT-1 might not be able to functionally replace GAT1 for the maintenance of physiological GABA levels, therefore pointing to a role of BGT-1 in brain tissues that differs from regulating GABA levels which might be linked to its ability to transport betaine and to a protective mechanism against osmotic stress. In addition, it was reported that a polymorphism in SLC6A12 can increase the risk of temporal lobe epilepsy (Sacher et al., 2002; Gonzalez-Burgos and Lewis, 2008; Kempson et al., 2014; Li et al., 2015).

## 4 Role of ions and substrates in transport function

### 4.1 Ion dependence

GAT1 and BGT-1 are both secondary active transporters, which exploit the inward-directed Na^+^ electrochemical gradient to energize the uphill transport of their substrate (Riggs et al., 1958; Kanner, 1978; Matskevitch et al., 1999; Hertz et al., 2013). In addition, Na^+^ ions also play a role in stabilizing the bound substrate inside the binding pocket of SLC6, leading to binding cooperativity between the co-transported ions and the organic substrate (Rosenberg and Kanner, 2008; Piscitelli et al., 2010). Replacing Na^+^ with other cations such as K^+^, Cs^+^, Li^+^, ammonium, and choline does not trigger GABA transport in GAT1, but Li^+^, and to some extent Cs^+^, can permeate the transporter in an uncoupled manner, leading to the leak-currents (Mager et al., 1996; Grossman and Nelson, 2003). GABA transport by GAT1 and BGT-1 is electrogenic, i.e., substrate uptake by both transporters leads to the net translocation of elementary positive charge(s) into the cell. As a consequence, the transport is voltage-dependent (Guastella et al., 1990; Matskevitch et al., 1999). Moreover, it has been observed that Cl^−^ ions are required for transport by GAT1 (Lu and Hilgemann, 1999a; Lu and Hilgemann, 1999b; Loo et al., 2000; Bossi et al., 2002; Giovannardi et al., 2003; Zomot et al., 2007) and BGT-1 (Yamauchi et al., 1992). The role of Cl^−^ ions remains incompletely understood since the equilibrium potential of Cl^−^ is close to the membrane resting potential, which means that the energetic contribution of co-transporting Cl^−^ does not contribute to the driving forces of the transport reaction (Loo et al., 2000). On the other hand, the presence of a negative charge in the binding site helps in stabilizing the Na^+^-bound conformation of the protein, facilitating transport (Zomot et al., 2007; Rosenberg and Kanner, 2008).

### 4.2 Kinetic parameters

For defining kinetic parameters of the transport reactions, two parameters are particularly important: the concentration of substrate (or co-substrate) necessary to reach the half maximum transport rate (in this work defined as K_0.5_), and the maximum turnover V_max_. Data on the apparent affinities of GAT1 and BGT-1 for their substrates have been collected using electrophysiological measurements and radioactive uptake assays (See Table 2). It is interesting to note that the K_0.5_ of GAT1 for GABA, which is in the low μM range, shows a two-fold difference when switching the membrane potential from −90 mV to −10 mV, while the same membrane potential modulation exerts a larger effect on K_0.5_ for Na^+^ and Cl^−^. For BGT-1, conversely, K_0.5_ of the organic substrates (μM range for GABA and low mM range for betaine) increases around four-folds, while the K_0.5_ for Na^+^ and Cl^−^ seems to be less affected by voltage changes. The V_max_ for GAT1 in the forward transport mode (GABA influx) is between 6 s^−1^ and 13 s^−1^ at voltages between −60 mV and −80 mV, at room temperature (between 20°C and 25°C), and can be influenced by modifying the temperature in *Xenopus laevis* oocytes; at 37°C and −50 mV or −90 mV the V_max_ is 73 s^−1^ and 93 s^−1^, respectively (Binda et al., 2002; Fesce et al., 2002; Bicho and Grewer, 2005; Gonzales et al., 2007). In the reverse transport mode (GABA efflux), the V_max_ is 3 s^−1^ at −120 mV and 60 s^−1^ at +120 mV (Lu and Hilgemann, 1999a).

**TABLE 2:** Apparent affinities of the transporters GAT1 and BGT-1 for the respective substrates with different techniques, samples, and conditions.

Transporter	Substrate	Technique	Condition	K_0.5_	References
rGAT1	GABA	[^3^H]GABA uptake in *X. laevis* oocytes	96 mM NaCl, 22.2 mCi [^3^H]GABA	7 µM	Guastella et al. (1990)
hGAT1	Na^+^	TEVC on *X. laevis* oocytes	−80 mV, 120 mM Cl^−^, 0.3 mM GABA	44 mM	Mager et al. (1996)
hGAT1	GABA	TEVC on *X. laevis* oocytes	−90 mV, 21°C, 100 mM Na^+^, 106 mM Cl^−^	34 µM	Gonzales et al. (2007)
			−10 mV, 21°C, 100 mM Na^+^, 106 mM Cl^−^	17 µM	
	Na^+^	TEVC on *X. laevis* oocytes	−90 mV, 21°C, 5 mM GABA, 106 mM Cl^−^	33 mM	
			−10 mV, 21°C, 5 mM GABA, 106 mM Cl^−^	108 mM	
	Cl^−^	TEVC on *X. laevis* oocytes	−90 mV, 21°C, 5 mM GABA, 100 mM Na^+^	9 mM	
			−10 mV, 21°C, 5 mM GABA, 100 mM Na^+^	88 mM	
mGAT2*	GABA	[^3^H]GABA uptake in *X. laevis* oocytes	100 mM NaCl, 0.1 µCi [^3^H]GABA	80 µM	Lopez-Corcuera et al. (1992)
hBGT-1	GABA	[^3^H]GABA uptake in HEK293 cells	140 mM NaCl, 2.76 mCi [^3^H]GABA	18 µM	Kvist et al. (2009)
cBGT-1	GABA	TEVC on *X. laevis* oocytes	−100 mV, 96 mM Na^+^, 103.6 mM Cl^−^	9 µM	Matskevitch et al. (1999)
			−30 mV, 96 mM Na^+^, 103.6 mM Cl^−^	31 µM	
	betaine	TEVC on *X. laevis* oocytes	−80 mV, 96 mM Na^+^, 103.6 mM Cl^−^	500 µM	
			−30 mV, 96 mM Na^+^, 103.6 mM Cl^−^	2 mM	
	Na^+^	TEVC on *X. laevis* oocytes	−90 mV, 1 mM GABA, 100 mM Cl^−^	66 mM	
			−50 mV, 1 mM GABA, 100 mM Cl^−^	89 mM	
	Cl^−^	TEVC on *X. laevis* oocytes	−90 mV, 1 mM GABA, 96 mM Na^+^	78 mM	
			−50 mV, 1 mM GABA, 96 mM Na^+^	114 mM	

^a^ mGAT2 is the mouse homologous of BGT-1 in dog, rat, and human (see Table 1).

### 4.3 Ion cooperativity

**FIGURE 2:**
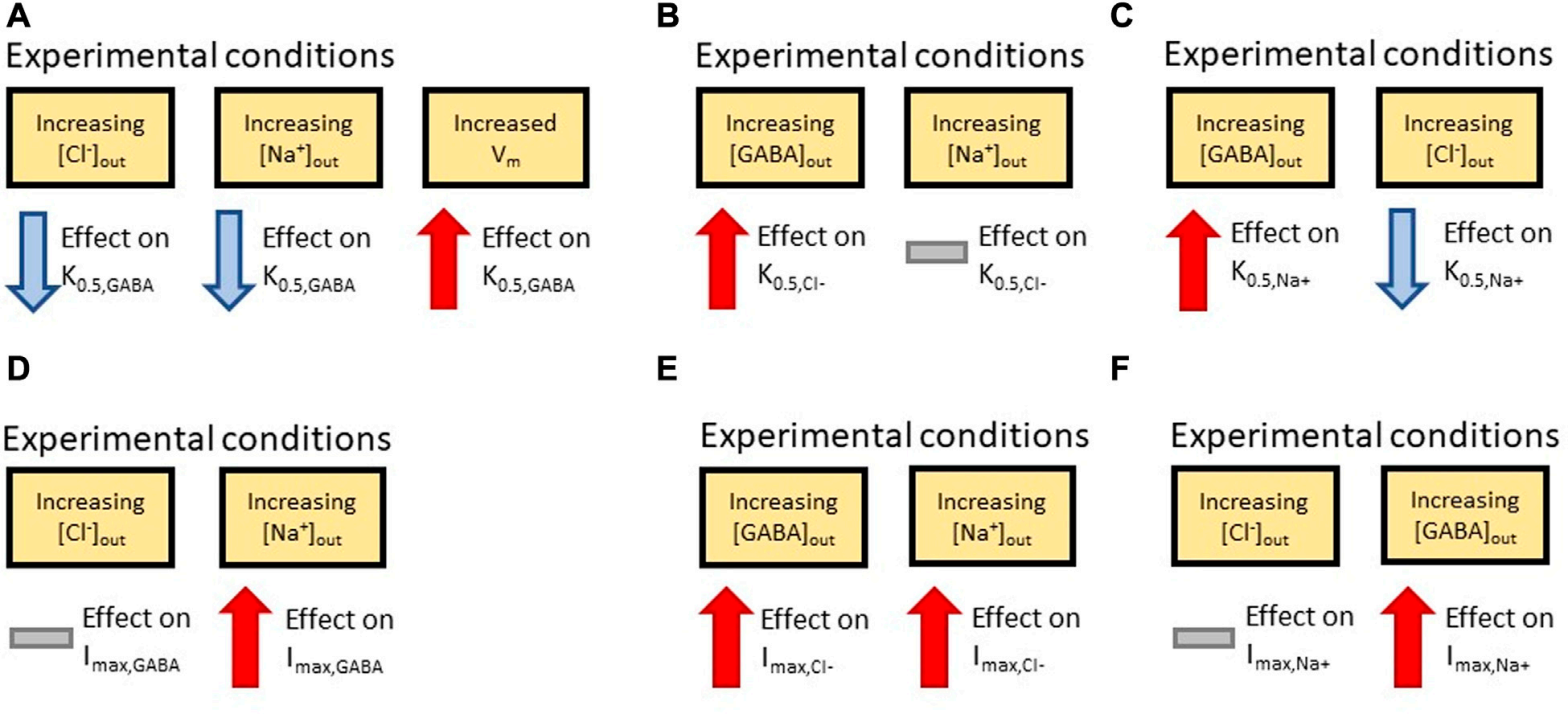
Changes in K_0.5_ or I_max_ as a consequence of the variation of experimental conditions; Figure is adapted from Loo et al. (2000). From **(A–F)**: The orange boxes represent changes in the experimental conditions, their effects on the parameter K_0.5_ or I_max_ are indicated below. A red upward arrow indicates an increase of the respective parameter, a blue downward arrow indicates a decrease, while a gray rectangle indicates no significant change.

Cooperativity is an effect of the modulation of the binding affinity for a substrate by the binding of other co-substrates. Since the binding affinity (K_d_) partially describes the kinetic K_0.5_, it is only natural to find that different concentrations of one substrate can exert an effect over the K_0.5_ of the other substrates. For GAT1, Loo and collaborators used two-electrode voltage clamp (TEVC) on *X. laevis* oocytes to study the cooperativity between Na^+^, Cl^−^, and GABA at −110 mV (Loo et al., 2000). The authors studied the cooperativity among substrates by considering the effect that a specific substrate extracellular concentration has on a kinetic parameter dependent on another substrate or voltage. The parameters considered are either the K_0.5_, or the V_max_ (proportional to the maximum current amplitude in TEVC, I_max_). From this study emerges that the apparent affinity for GABA is highly dependent on both external Na^+^ and Cl^−^ concentrations. The half-maximal GABA concentration (K_0.5, GABA_) decreases several folds when increasing external Na^+^ and Cl^−^ concentrations, while it increases dramatically when holding the voltage to a more positive value (See Figure 2A). This is expected since, as stated in Section 4.1, Na^+^ is needed to energize the transport and it is also possible that it can help stabilize the substrate in the binding pocket. Moving on Cl^−^, the half-maximal Cl^−^ concentration (K_0.5, Cl_^−^) increases upon increasing the extracellular GABA concentration, while no significant differences have been noted when altering external Na^+^ concentrations (See Figure 2B). Concerning Na^+^, the half-maximal Na^+^ concentration (K_0.5, Na_^+^) shows a slight increase when increasing extracellular GABA and a slight reduction when increasing extracellular Cl^−^ (See Figure 2C). The maximal current driven by saturating GABA (I_max, GABA_) seems not to be significantly altered by external Cl^−^ concentrations, while it increases significantly when increasing the extracellular Na^+^ concentration (See Figure 2D). Transport currents in presence of saturating external Cl^−^ (I_max, Cl_^−^) increase when increasing both external GABA and Na^+^ (See Figure 2E), while the transport currents in presence of saturating extracellular Na^+^ (I_max, Na_^+^) are unaffected by changes in extracellular Cl^−^ concentrations but increase dramatically when increasing external GABA (See Figure 2F).

### 4.4 Effect of Cl^−^ ions on the transport activity

When changing the membrane potential, the GABA-evoked current in the absence of Cl^−^ is greatly reduced when the holding potential is set to 0 mV, while at −150 mV the current is half of the amplitude of that obtained in the presence of 106 mM Cl^−^ (Loo et al., 2000). This is consistent with the idea that the main role of Cl^−^ is to attract positive charges in the binding site of GAT1, thus facilitating transport. Consistently, it is easier for positive charges (Na^+^) to access to the substrate and ion binding site in the center of the protein at highly negative membrane potential compensating for the absent Cl^−^ (Rosenberg and Kanner, 2008). A study conducted on the mutants S331E and S331D (Zomot et al., 2007) supports this hypothesis. Residue S331, which is involved in chloride binding, is substituted by these mutations with a negatively charged amino acid. The presence of this negative charge can, to some extent, substitute for the negative charge of the chloride ion, and the mutants partially restore transport in the complete absence of chloride anions. Similar to GAT1, also for BGT-1 the transport of betaine and GABA is dependent on Na^+^, and only partially dependent on Cl^−^. As for GAT1, hyperpolarization of the membrane increases affinity for Na^+^ in BGT-1. The reduction of external Na^+^ implies a decrease in both the maximal current and the affinity of the transporter for GABA. The dependence is mutual: decreasing the concentration of GABA below its K_0.5_ leads to a shift in K_0.5_ of Na^+^ from 50 mM to 120 mM. As for GAT1, GABA transport is partially dependent on Cl^−^, with currents decreasing by 80% at −90 mV and by 90% at −50 mV in the absence of Cl^−^. This implies that the Cl^−^ dependence is related to membrane polarization. Reducing the Cl^−^ concentration from 150 mM to 50 mM brought a slight decrease in GABA affinity. The affinity of Na^+^ and Cl^−^ is also mutually dependent: decreasing Cl^−^ brings a slight reduction in Na^+^ affinity, while the same reduction in Na^+^ leads to a non-saturable Cl^−^ concentration dependence (Matskevitch et al., 1999).

### 4.5 The reverse transport mode

The reverse transport phenomenon triggers the release of GABA in the synaptic cleft through GAT1 or BGT-1, as well as the release of betaine through BGT-1. The reverse transport remains understudied in both transporters, while the role of substrate concentration in regulating the ratio between reverse and forward transport remains largely unknown due to the difficulties in controlling cytoplasmic concentrations. For GAT1, a number of interesting results have been described. The reported ratio between the forward and the reverse transport mode is tuned by membrane potential, if no specific conditions are applied to intra- and extra-cellular substrates concentrations: at negative potentials, forward transport is favored, while the equilibrium is shifted towards reverse transport with membrane depolarization (Wu et al., 2001). This observation has biological relevance as the membrane potential reaches a positive value when the neuron is firing at an action potential. The work of Loo and collaborators show that altering the Na^+^ gradient can regulate the influx or efflux of GABA (Wu et al., 2006). If Na^+^ is present only inside the cell, this results in a net, albeit small outward-directed GABA-evoked current. The results were more complex, when extracellular GABA was removed: a GABA-evoked outward-directed current was recorded followed by an inward-directed current that was attributed to GABA being reabsorbed by the cell. The effect, though, is stronger when altering the Na^+^ gradient as compared than changing the GABA gradient due to the stoichiometry of the transport reaction. This indicates a possible regulatory role in a physiological environment: once a neuron has fired a series of action potentials with high frequency the internal sodium concentration may become sufficiently high to allow for limited release of GABA to the synaptic cleft (Wu et al., 2006) or to reduce the uptake of synaptically released sodium. Similarly reducing the cytoplasmic concentration of Cl^−^ favors GABA uptake, while high intracellular Cl^−^ promotes reverse transport (Grossman and Nelson, 2003). Consistently, Bertram and collaborators showed that in the presence of internal GABA, the depletion of internal Cl^−^ in *X. laevis* oocytes expressing GAT1 resulted in a significant decrease of reverse transport activity (Bertram et al., 2011).

### 4.6 Effect of Na^+^ and Cl^−^ on pre-steady state current

In electrophysiology, the continuous transport activity by an electrogenic transporter is registered as the elicitation of a steady state current. The electrophysiological measurements, apart from the steady-state currents, also let to record an exponentially decaying transient current, known as the pre-steady state current. They represent the intramembrane charge movement seen as a transient current in response to voltage or Na^+^ concentration jumps and can be recorded by voltage clamp in *X. laevis* oocytes (Bhatt et al., 2022) and in HEK cells (Bicho and Grewer, 2005). These currents are associated with the initially synchronized out-of-equilibrium events such as binding and release of ions, conformational rearrangements, and charge movements across the membrane electric fields (Wadiche et al., 1995; Peres et al., 2004). For the GABA transporter rGAT1, the charge movement is related to the displacement of ions (Na^+^) between the extracellular space and a cavity in the transporter. The analysis of these currents provides kinetic parameters such as rate constants and charge dislocation during the pre-steady state event (Holmgren and Rakowski, 1994). Using voltage clamp protocols in *X. laevis* oocytes expressing transport protein it was possible to isolate pre-steady state currents elicited by GAT1 and BGT-1 (Mager et al., 1993; Mager et al., 1996; Bossi et al., 2002; Grossman and Nelson, 2003). These transient currents in GAT1 and BGT-1 are strictly Na^+^-dependent and reflect the movement of Na^+^ in the transporter vestibule inside the membrane electric field for the initial step of the transport. For GAT1, this charge relocation accounts for most of the total charge transferred per transport cycle (Bicho and Grewer, 2005; Lopez-Redondo et al., 2018). Interestingly, the pre-steady state currents in BGT-1 and GAT1 are different. In GAT1, the Na^+^ induced transient currents are symmetric at both positive and negative membrane potentials. Whereas in BGT-1 the Na^+^-induced pre-steady state currents are only detectable at negative membrane potentials. This behavior can be modified by modulating external Na^+^ concentrations for both BGT-1 and GAT1: if extracellular Na^+^ is lower than its K_0.5_ for the respective transporter, the pre-steady state currents are detected only at negative membrane potentials, while if extracellular Na^+^ is several folds higher than its K_0.5_, these currents were detected only at positive potentials. These results suggest that the pre-steady state current results from the exposure and the occlusion of charges on the transporter surface (Forlani et al., 2001; Binda et al., 2002; Grossman and Nelson, 2003). For GAT1, reducing the concentration of external Na^+^ and/or Cl^−^ would shift the charge versus voltage relation of the transient currents towards more negative potentials, where the effect of Cl^−^ is less than Na^+^. In GAT1, the transient currents are still observable in the absence of external Cl^−^ indicating an only partial dependence of transport on extracellular Cl^−^ (Bossi et al., 2002).

### 4.7 pH dependence

Another factor that influences transport is pH. In BGT-1, the current elicited by GABA decreases with decreasing pH (at pH 5.5, the current reduces to 20% with respect to the current at physiological pH) and strongly increases at pH 8.5. In contrast, for GAT1 the GABA induced currents decrease only slightly at acidic pH, while no change was observed at alkaline pH (Forlani et al., 2001). For both transporters, acidification combined with a decrease in Na^+^ reduces currents much stronger than by the sole decrease in Na^+^. In BGT-1, the change in pH does not significantly alter the K_0.5_ for GABA and Na^+^. In contrast, the Cl^−^ K_0.5_ is highly affected by pH: it is 50 mM at pH 8.5, but seemingly non-saturable at pH 7.0 (Matskevitch et al., 1999). The pre-steady state currents of GAT1 are affected by alkaline pH: while present at both positive and negative potentials with similar absolute amplitudes, at alkaline pH the currents are measurable almost exclusively at positive potentials (Grossman and Nelson, 2003). No pre-steady state current is detectable for BGT-1 at acidic pH, while at pH 8.5 they can be observed at positive potentials (Matskevitch et al., 1999). An interpretation of these observations could be that H^+^ could compete with Na^+^ for the sodium binding sites in both GAT1 and BGT-1, but not facilitate a substrate transport (Matskevitch et al., 1999; Forlani et al., 2001; Binda et al., 2002). In GAT1 it has been reported that one of the determinants of the pH dependence is located in external loop 5 (EL5) (Forlani et al., 2001).

### 4.8 Substrate stoichiometry

There are still controversies regarding the stoichiometry of the transport reaction for GAT1. Initial studies on membrane vesicles and proteoliposomes from rat brain slices pointed to a substrate stoichiometry of 2 Na^+^: 1 Cl^-^: 1 GABA, which was confirmed by the measurements of reversal potential in *X. laevis* oocytes heterologously expressing GAT1 (Radian and Kanner, 1983; Keynan and Kanner, 1988; Lu and Hilgemann, 1999b). Other authors observed a charge translocation that exceeds the before mentioned predicted stoichiometry and attributed the observation to a “channel-mode” behavior of GAT1 (Krause and Schwarz, 2005). This has been contested by others, who could not observe the same behavior in the same cell types (Matthews et al., 2009). Lately, a stoichiometry of 3 Na^+^: 1 Cl^-^: 1 GABA has been proposed from substrate uptake data and reversal potential measurements in *X. laevis* oocytes, because the reported data accounted by modelling of the experimentally observed elementary charges translocation could be explained better by the modified stoichiometry (Mager et al., 1996; Omoto et al., 2012; Willford et al., 2015; Eskandari et al., 2017). The importance of Cl^−^ for substrate transport is well documented (See Section 4.4 and Section 4.6) but its role remains debated. Some authors suggested a Cl^−^/Cl^−^ exchange mechanism for GAT1, suggesting no occurrence of the net Cl^−^ transport, and proposed that the net stoichiometry for the transport cycle should be 2 Na^+^: 1 GABA (Loo et al., 2000; Bicho and Grewer, 2005). For BGT-1 only one transport stoichiometry has been reported. Matskevitch and collaborators measured radioligand uptake in *X. laevis* oocytes and reported that two ratios would be possible: 3 Na^+^: 1 or 2 Cl^−^: 1 GABA/betaine (Matskevitch et al., 1999).

## 5 GABA transporter structure

### 5.1 Early studies of structural elements

Initially, structures of bacterial homologues of the human SLC6 family, like LeuT in *Aquifex aeolicus* or MhsT transporter in *Bacillus halodurans* were resolved by crystallography followed by the first structures of transporter from eukaryotic organism: the biogenic amine transporter, the *Drosophila melanogaster* dopamine transporter (dDAT), and the human serotonin transporter (hSERT) (Yamashita et al., 2005; Penmatsa et al., 2013; Coleman et al., 2016). More recently, using cryo-electron microscopy (cryo-EM), a number of mammalian transporters from the SLC6 family have been determined, including the substrate-bound SERT, the glycine transporter 1 GlyT1, the neutral amino acid transporter B^0^AT1 and the GABA transporter 1 GAT1 (Coleman et al., 2019; Shahsavar et al., 2021; Joseph et al., 2022; Motiwala et al., 2022). While the structure of BGT-1 is still not known, we can obtain insights from these existing structural models, especially GAT1 given that they belong to the same sub-family.

**FIGURE 3:**
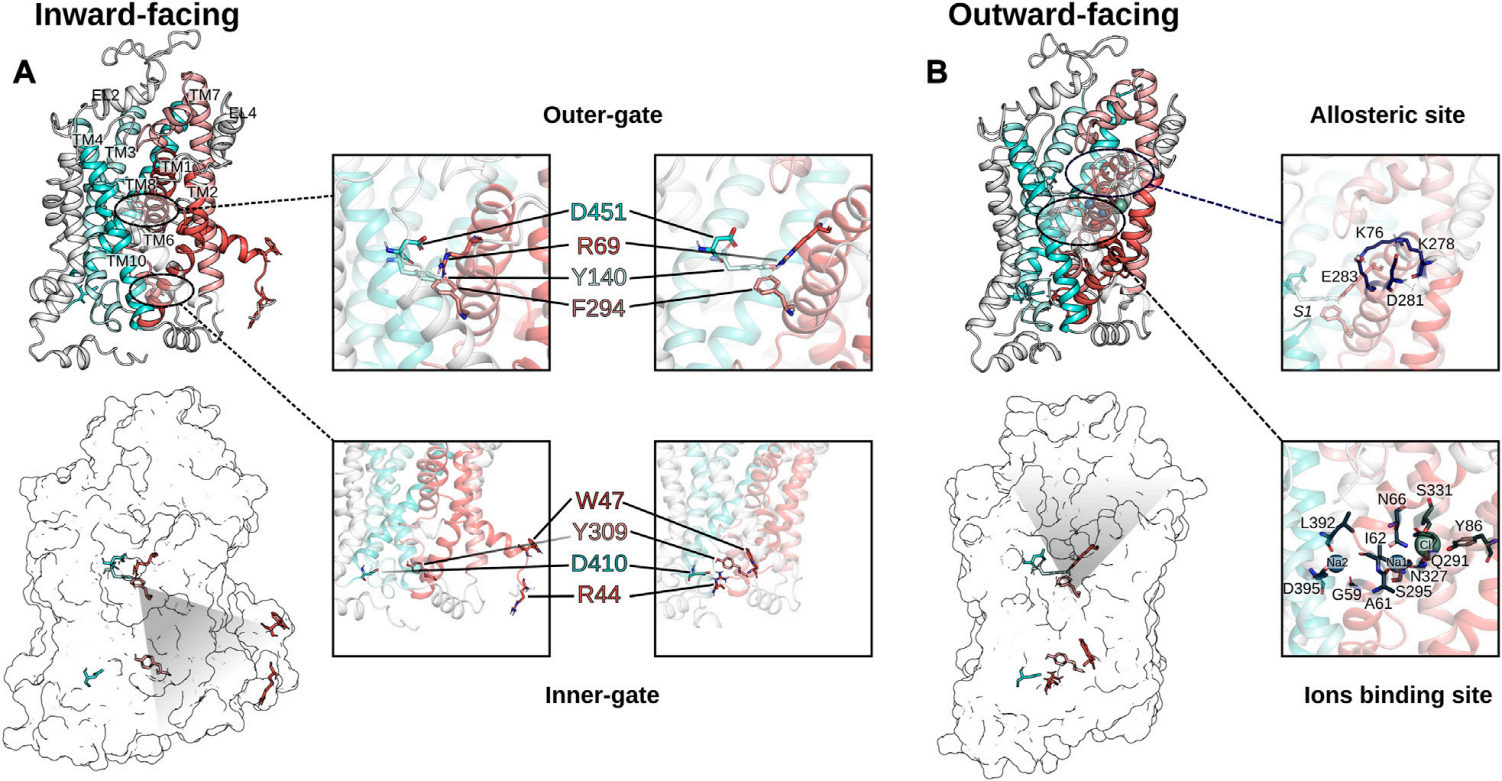
Comparison of the inward-facing conformation **(A)** and the outward facing conformation **(B)** of GAT1. The inward-open and the outward-open conformations of GAT1 are shown in secondary structure rendering to visualize the TM helices (scaffold domain in cyan colors, bundle domain using red colors) and as transparent surface rendering to highlight the residues of the extracellular gate and the intracellular gate. The inserts zoom-in on the outer and the inner gate to highlight the structural differences of the two conformations. On the right of **(B)** are shown two zoom-in inserted on the allosteric site S2 and on the ion binding residues, respectively.

Despite the low sequence homology between LeuT and the human homologues (20%–25%), the fold (LeuT fold), the transport mechanism, and several residues essential for function are conserved. The LeuT fold displays an inverted pseudo-twofold symmetry comprising 12 transmembrane helices (TM), two sodium binding sites (Na1 and Na2), the substrate binding site S1, and an allosteric site S2 (Yamashita et al., 2005; Forrest et al., 2007) as indicated in Figure 3. The scaffold domain is composed of TM3, 4, 8, 9, and the first intracellular loop. The SLC6 transporters have two dominant conformations, which strikingly differ in their accessibility to the substrate binding site S1 that is located halfway through the membrane. In the outward-facing conformation, the S1 can be reached only from the extracellular side through the open extracellular vestibule, while in the inward-facing conformation, the outer vestibule is sealed and the S1 can be reached by the open intracellular vestibule. The transport cycle of SLC6 transporters can be described by the alternating access model (Jardetzky, 1966), where the transporter cycles between the outward-facing conformation that is ready for binding substrate from the extracellular side and the inward-facing conformation that releases substrate to the cytosol. These structural changes are accomplished by motions of the bundle domain. It comprises TM1, 2, 6, 7, and the connecting intracellular (IL) and extracellular loops (EL). TM1 and 6 have an unwound section halfway across the membrane (Forrest et al., 2007). These two discontinuous helices (TM1a/b and TM6a/b) expose backbone atoms involved in ion and substrate binding to create each a dipole that contributes to tight binding of the substrate (Yamashita et al., 2005; Gradisch et al., 2022). Therefore, the centrally located TM1 and 6 form the core of the transporters and prominently contribute to the substrate and sodium binding sites. While the non-transmembrane N and C-terminals are not essential for function, the ELs and ILs are crucial structural elements for structural and functional integrity (Pantanowitz et al., 1993; Kanner et al., 1994; Tamura et al., 1995; Zomot and Kanner, 2003; Rosenberg and Kanner, 2008; Ben-Yona and Kanner, 2009; Dayan et al., 2017; Dayan-Alon and Kanner, 2019).

**FIGURE 4:**
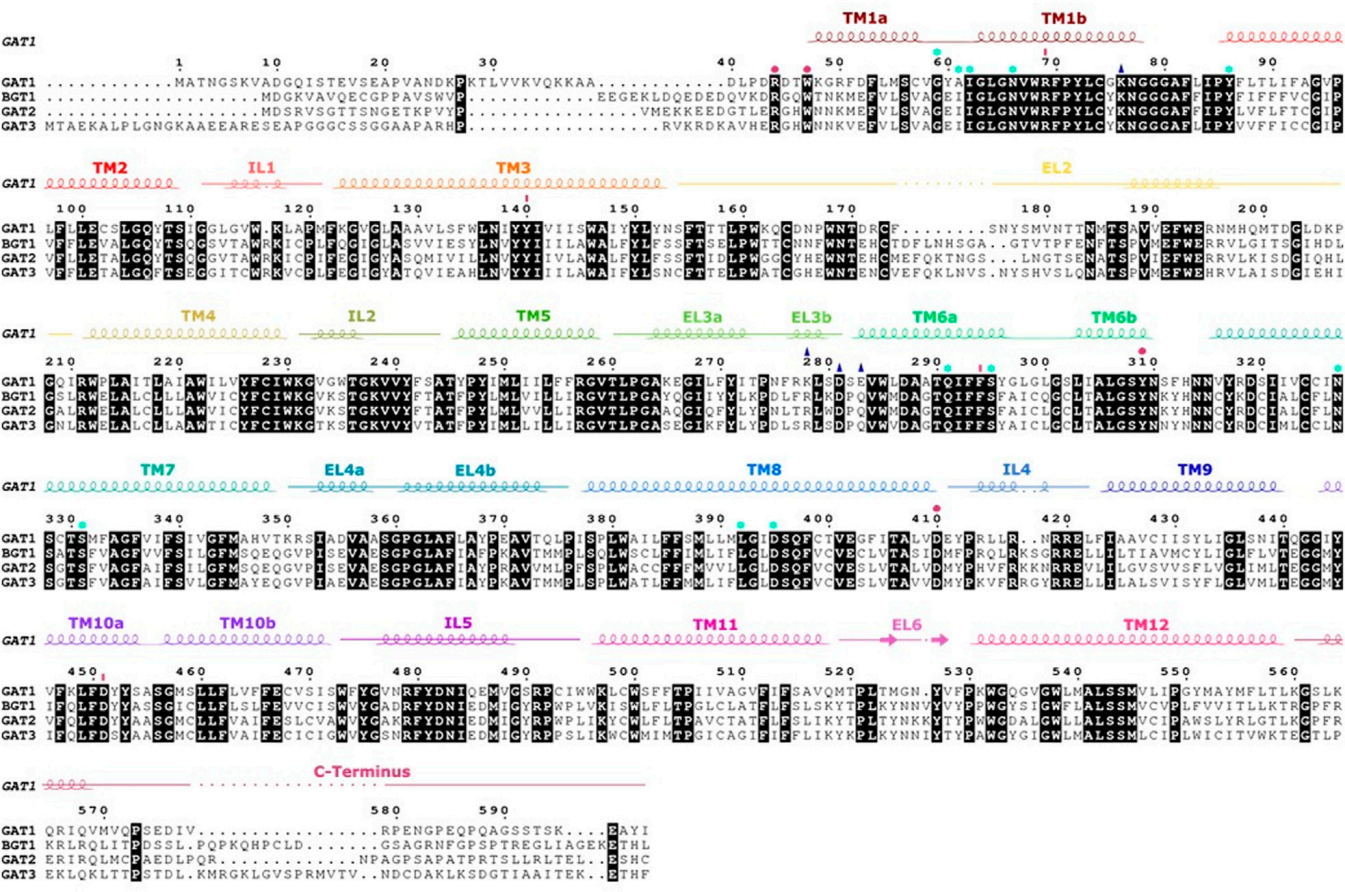
Sequence alignment and secondary structure of the four human GATs. Strictly conserved residues are highlighted in black. The α-helices and β-strands in hGAT1 are shown as coils and arrows, respectively, above the alignment as present in the structure of human GAT1 [PDB ID: 7SK2 (Motiwala et al., 2022)]. Intracellular and extracellular regions are indicated by a strike-through line. Amino acids involved in the inner and outer gates are symbolized by pink dots and lines, residues interacting with Na^+^ and Cl^−^ are represented by light blue stars. The amino acids of the allosteric site are shown with a dark blue triangle.

Several GAT1 mutations in TM1, 6, 10, and EL4 and 5 were assessed for their impact on transporter function. The ELs were initially studied by residue deletions from EL4 and EL5, which resulted in less than 2% of GAT1 wild-type activity (Kanner et al., 1994). Also, the chemical modification of the GAT1 EL4 cysteine mutant (A364C) by sulfhydryl reagents affected transport function: no steady-state current in the presence of Na^+^ and GABA could be measured, suggesting that the chemical modification of A364C blocks the conformational changes required during GABA transport. In contrast, pre steady-state current in the presence of Na^+^ remained unchanged, showing that Na^+^ and GABA binding were unaffected (Zomot and Kanner, 2003). It was observed that the chemical modification of A364C increased the accessibility of residues in the inner vestibule (C399, E402, T406, and D410), supporting the notion of affecting the conformational equilibrium and stabilizing an inward-facing conformation by opening the cytoplasmic pathway to the S1 (Ben-Yona and Kanner, 2009). The S1 can be accessible from the extracellular side or the intracellular side through an open passage, but at least one of the two passages is closed at any time by a gate, each supported by an intramolecular salt bridge across the passage. The extracellular gate involves residues from TM1b, 6a, 10, and EL4, 5. Sequence alignments showed that TM10 of GABA transporters carries an additional residue as compared to the other SLC6 transporters, which might form a π-helix element and represent a flexing point that is important for the conformational changes of the transport cycle (see Figure 4). Individual deletion of one residue (ΔS456, ΔG457 and ΔM458) from this π-helix element in TM10 resulted in loss of function mutants and constitutive leak currents (Dayan et al., 2017). TM1 was extensively studied as it is a versatile transmembrane helix involved in intracellular and extracellular gating and is important for the switch from a leaking state to a transport-competent conformation (Yu et al., 1998; Zhou et al., 2004; Ben-Yona and Kanner, 2013). The Y60C mutant induces leak currents in the presence of Na^+^, suggesting that Y60 is involved in sodium affinity or in closing of the inner gate (Kanner, 2003). The modification of the G63C mutant with sulfhydryl reagent blocked transient currents typically induced by lithium. The conserved tryptophan (W68 in GAT1) in TM1 is essential for structural activity (Zhou et al., 2004). Its mutation leads to a loss of expression or function in all SLC6 transporters. In GAT1, conservative substitution of W68 with another aromatic residue resulted in a loss of surface expression and function (Kleinberger-Doron and Kanner, 1994). Residue R69 (TM1) is part of the salt bridge with D451 (TM10) of the outer gate and one of the five charged residues located in the transmembrane region. A conservative R69K mutation or the double mutant R69K and D451E blocked transport activity (Pantanowitz et al., 1993; Ben-Yona and Kanner, 2013). At the intracellular gate, the symmetry related salt bridge of the inner gate is formed by R44 (TM1) and D410 (TM8). The rGAT1 mutants R44H and D410E retained GABA transport activity, but their function was impaired, while single mutant R44K showed a reduced affinity for sodium, thereby suggesting that these mutants altered conformational coupling and/or the conformational equilibrium (Bennett et al., 2000; Ben-Yona and Kanner, 2013; Dayan-Alon and Kanner, 2019). Consistent with this interpretation, a double mutant of both aspartate residues (inner gate D410E and outer gate D451E) to glutamate restored transport activity of the respective transport deficient single mutants of GAT1 (Dayan-Alon and Kanner, 2019). Similar to TM1, early data on the reaction of the cysteine mutants of W285C, L286C, D287C, S295C on TM6a with sulfhydryl reagent indicated that these residues should line the access path from the extracellular space to the S1 as the presence of GABA decreased their reactivity with sulfhydryl reagents (Rosenberg and Kanner, 2008).

### 5.2 Na^+^ and Cl^−^ binding sites

Most human SLC6 are chloride dependent, with the exception of B^0^AT1 and B^0^AT2 (Broer et al., 2006; Margheritis et al., 2013), while also LeuT is chloride independent. The chloride binding site was independently identified by Zomot and collaborators by comparing LeuT with GAT1 (Zomot et al., 2007) and by Forrest and collaborators (Forrest et al., 2007) in SERT. In GAT1, Zomot and collaborators identified residues Y86, Q291, S295, N327, S328 and S331 to constitute the putative chloride binding site. In designing chloride-independent mutants they established that the S331E mutant functionally replaces the chloride in GAT1, while in LeuT, the corresponding residue E290 is essential for chloride independent transport (Ben-Yona et al., 2011). Similarly by a comparison with LeuT, Forrest and collaborators (Forrest et al., 2007) identified the chloride binding site in SERT by substituting S372 with D or E, thereby rendering transport in SERT chloride independent (Forrest et al., 2008). This chloride binding site could later be confirmed by structure determination of the dDAT (Penmatsa et al., 2013). These results established that the negative charge next to the Na1 binding site is essential for transport. While in the chloride independent SLC6 transporters the charge is introduced by a glutamate in TM7 in the position corresponding to residue E290 of LeuT, in the human SLC6 transporters, this position is in most cases a serine (i.e., S331 in GAT1 or S372 in SERT) and predisposes the transporter for chloride binding. The exception is NTT5, where the serine is a leucine (see Figure 4). Interestingly, B^0^AT1 and B^0^AT2 also harbor a serine in this position. It was shown that these two transporters have a higher chloride affinity, and it was therefore suggested that chloride remains continuously bound (Broer et al., 2006).

Q291 in GAT1 is a residue that is highly conserved in the eukaryotic NSS transporter and the bacterial homologues. It is located in TM6 and is in direct contact with the chloride ion. Replacing Q291 in GAT1 with smaller or acidic side chains, it was shown that this residue plays an important role in ion binding, substrate transport and currents (Mari et al., 2006; Ben-Yona et al., 2011). The importance of this residue for the transport cycle through its role in coordinating the sodium-stabilizing chloride ion is highlighted by the complete loss of detectable GABA uptake activity and the strongly altered voltage dependence of the GABA-induced steady state current in the Q291S and Q291N mutants (Ben-Yona et al., 2011). While it has been established that Q291 is involved in chloride coordination, Ben-Yona et al. (2011) inferred a second role for Q291 from the similarities in the increased apparent sodium affinity of Q291N and of R69K, suggesting that R69 and Q291 directly interact during the transport cycle. This suggestion was confirmed by the recent cryo-EM structure of GAT1 (Motiwala et al., 2022).

Cation binding to the Na1 and Na2 of GAT1 and their kinetics have been studied in detail and are described in Section 4 (MacAulay et al., 2002; Mari et al., 2006; Zhou et al., 2006; Meinild and Forster, 2012). Sodium binding to GAT1 was shown to precede GABA translocation (Radian and Kanner, 1983; Fesce et al., 2002; Bicho and Grewer, 2005). Kinetic modeling of GABA transport derived from electrophysiological data indicated that the sodium affinities to Na1 and Na2 of GAT1 differ ∼100 fold, with a K_d_ of 10 mM for the high affinity side and 920 mM for the low affinity side (Hilgemann and Lu, 1999). In contrast to sodium, lithium does not support transport activity or induce pre-steady state currents. It has been suggested that the lithium-bound state is structurally distinct from the sodium-bound state (MacAulay et al., 2002), as leak currents were detected in the presence of lithium, which can be blocked by low concentrations of Na^+^. This is not due to a competition between the two cations, but because of a switch from a leak current state to the sodium-stabilized outward-open conformation (Zhou et al., 2006). The structure of LeuT allowed to identify the residues contributing to the Na1 and Na2 sites in GAT1 through sequence comparison: residues N66, N327 and S295 coordinate sodium in the Na1 site, while D395 and S396 coordinate sodium in the Na2 site (Zhou et al., 2006). These predictions through modelling were experimentally verified. In the presence of 150 mM sodium, the transport velocity of the N327A and N327C variants was reduced by 20 to 80 folds. Transport of GABA could still be observed, but required higher concentrations of sodium (Zhou et al., 2006), showing that the GAT1 N327 variants remained sensitive to sodium despite mutation of the Na1 site. The Na2 site is considered to be more promiscuous, because a D395T or D395C mutant maintained coupled GABA currents and interactions with lithium (Zhou et al., 2006). Another residue in GAT1 that was studied for its role in sodium binding and GABA transport is E101 (Keshet et al., 1995). This residue is conserved in the SLC6 family and corresponds to E62 in LeuT, E66 in MhsT, and E136 in SERT (See Figures 3, 4). In GAT1, even the conservative mutant E101D reduced transport activity to 1% (Keshet et al., 1995), while its mutation in SERT (E136D) reduces transport to a lesser extent, while any other mutant abolished transport (Korkhov et al., 2006). Structures of SLC6 transporters showed later that E101 does not directly interact with sodium ions, it is not exposed to the substrate binding site S1 or the permeation path through the transporter. This conserved glutamate residue can be considered a structurally and mechanistically essential residue that is associated with maintaining structural integrity, most importantly of the unwound region of TM6.

### 5.3 Substrate binding sites

Sodium binding is the first step of the transport cycle, because the high extracellular NaCl concentration leads to fast binding. The outward-facing state is stabilized by sodium binding and the affinity of the substrate increases strongly with the presence of Na^+^ (Keshet et al., 1995; Mager et al., 1996; Lu and Hilgemann, 1999a; Lu and Hilgemann, 1999b; Kanner, 2003; Szollosi and Stockner, 2021; Szollosi and Stockner, 2022).

Sequence comparison revealed that the composition of the six ELs in GAT1 are significantly different compared to the three other GABA transporters (See Figure 4). Respective amino acid substitutions resulted in a change in affinity towards GABA, β-alanine, or taurine (Tamura et al., 1995). In fact, the ELs interact with inhibitors and maintain a conformation that allows the substrate to access the substrate binding site. Inhibitors were found to bind to the orthosteric substrate binding site S1 or to the allosteric binding site S2 site located in the outer vestibule (See Figure 4). Crystallization of LeuT with tricyclic antidepressant drugs (TCA) showed that D401 (EL5) interacted with the nitrogen atom of the drug through a salt bridge (Singh et al., 2007; Zhou et al., 2007; Wang et al., 2013). This is supported by the impact of TCAs on the net currents of the GAT1 K448E mutant, which is equivalent to the residue of D401 in LeuT and emphasizes the role of this residue in GAT1 for the interaction with inhibitors (Cherubino et al., 2009). Consistently, several inhibitors of SERT and LeuT were found to bind to S2 (Singh et al., 2007; Quick et al., 2009; Wang et al., 2013; Coleman et al., 2016; Erlendsson et al., 2017).

The recent structure of GAT1 (Motiwala et al., 2022) showed that G63 (TM1 unwounded region) and S295 (TM6a) coordinate the carboxyl group of the inhibitor, and suggested coordinating also the carboxyl group of GABA, confirming earlier results indicating that G63 and S295 are involved in GABA binding and transport (Kanner, 2003; Rosenberg and Kanner, 2008). Three other residues predicted in earlier studies to interact with GABA are G65 and L64 on TM1b and Y140 on TM3 could be confirmed (Bismuth et al., 1997; Mari et al., 2006). Interestingly, the saturation uptake assay of GAT1 in the presence of tiagabine suggested a two steps mechanism leading to GAT1 inhibition, whereby the first step is consistent with a competitive inhibition followed by a transition to a non-competitive inhibition. If confirmed and generalizable, the inhibition mechanism would resemble the binding of ibogaine (Coleman et al., 2019) at the monoamine transporters and indicate that also other non-competitive inhibitors might use this mode of binding (Cherubino et al., 2009). For the monoamine transporters, this mode of binding has been observed for ligands that were shown to have the potential for rescuing folding deficient transporter mutants (Freissmuth et al., 2018). It raises the importance of pharmacology research on GABA transporters to identify compounds able to rescue disease-causing folding deficient GATs.

### 5.4 Oligomeric state

SLC6 transporters can form oligomers, whereby the dimerization interface is limited to the scaffold domain, but not conserved: for example, in LeuT it involves TM9 and the structurally non-conserved TM12, in the bacterial Na^+^/H^+^ exchanger NhaA it consists of most TM helices of the scaffold domain and is stabilized by a non-conserved additional β-sheet structure (Yamashita et al., 2005; Lee et al., 2014). The oligomeric state and its role for human SLC6 transporters is still under discussion, while the functional unit seems to be the transporter monomer. GlyT1 and GlyT2 are monomeric, whereas hSERT shows a range of oligomeric states (monomer to hexamers) that is in part controlled by the concentration of the signaling lipid phosphatidylinositol-4,5-biphosphate (PIP_2_) (Horiuchi et al., 2001; Anderluh et al., 2017). Similarly, DAT and NET show PIP_2_ dependent formation of dimers (Das et al., 2019). On SDS-PAGE, GAT1 is visible as a dimer and more broadly an oligomer, but similar to the monoamine transporters its functional unit is the monomer (Farhan et al., 2004; Soragna et al., 2005; Mari et al., 2006). The oligomeric state of the NSS is important for surface expression, but it seems to play no direct role in the function of cellular substrate uptake (Mari et al., 2006; Anderluh et al., 2017; Jayaraman et al., 2018; Das et al., 2019; Jayaraman et al., 2021). Oligomerization simulations of the DAT have shown that the interface between SLC6 transporter in the oligomers is confined to the scaffold domain, while sparing the transport domain, as no interactions involving this domain were observed (Jayaraman et al., 2018). This can be rationalized by the fact that the bundle domain needs to move during the transport cycle. Any interaction involving this domain would therefore at least decelerate, if not completely arrest the transport cycle.

### 5.5 Pharmacology of GATs

GAT1 is a well-recognized therapeutic target for the treatment of neurological disorders that are linked to a dysregulation of GABA homeostasis. The GABAergic system is known for its inhibitory role in the CNS and the periphery (Kanner, 2003). The physiological levels of GABA, and therefore brain homeostasis, are maintained by GATs through the rapid reuptake of the synaptically released GABA into the presynaptic neuron and glial cells. The pharmacological purpose of targeting GATs by inhibition is to bring excitatory/inhibitory balance in the CNS by increasing the GABA concentration in the synaptic cleft providing an increased inhibitory neurotransmission. To date, GAT inhibitors are used for the treatment of epilepsy, depression, and anxiety (Schousboe et al., 2004; Schousboe et al., 2017; Schousboe and Madsen, 2017).

**FIGURE 5:**
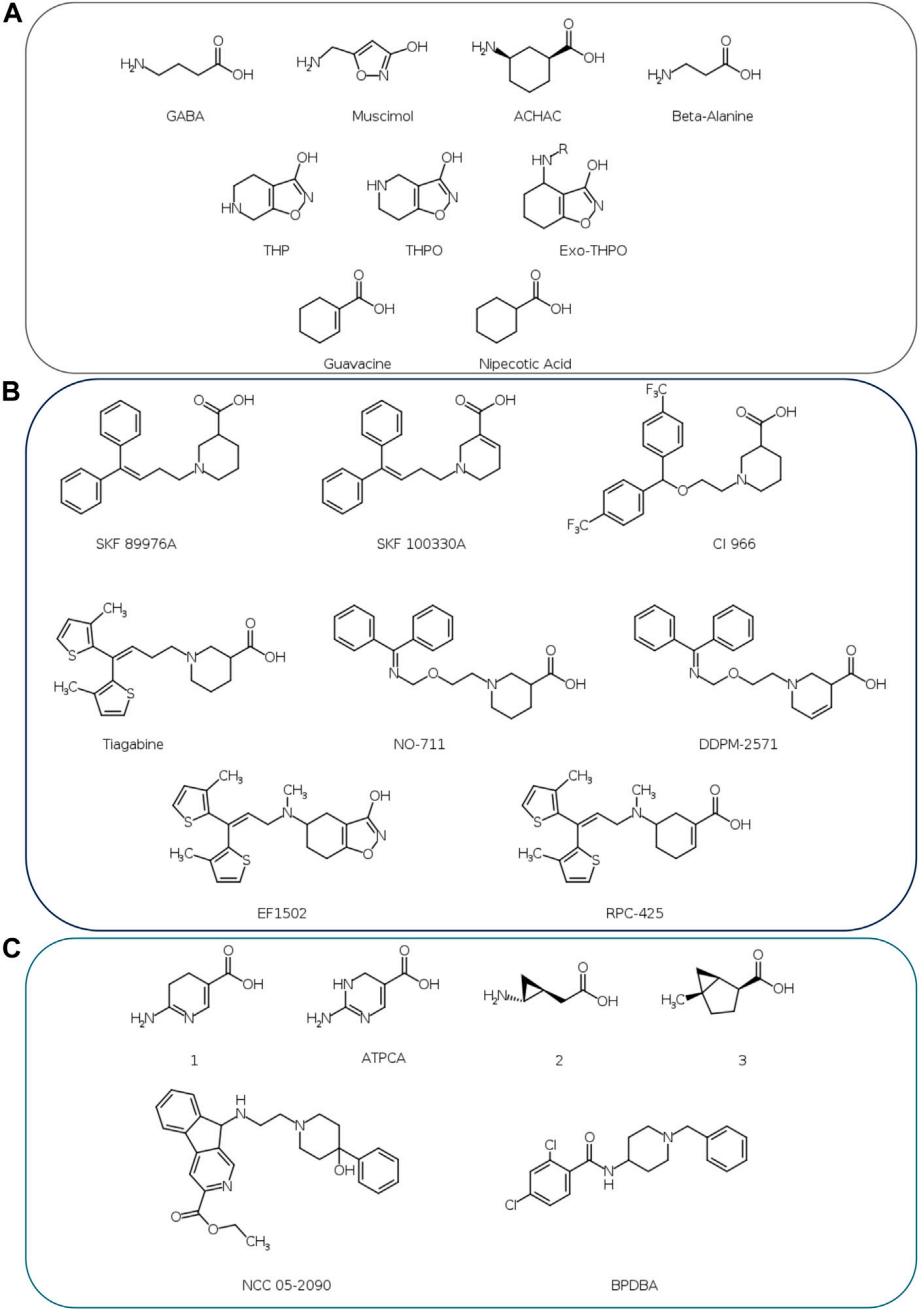
**(A)** Chemical structures of GABA and its analogues. These analogues show affinity to GATs and were used as starting points for developing competitive inhibitors. **(B)** Molecular structures of potent GAT1 inhibitors. **(C)** Molecular structures BGT-1 inhibitors.

The first step towards the characterization of the structure-activity relationship for GATs started with the discovery of Muscimol, a natural alkaloid, present in the mushroom *Amanita muscaria,* that could act as a potent GABA_A_ agonist and uptake inhibitor (Krogsgaard-Larsen et al., 1979; Krogsgaard-Larsen et al., 1981; Fariello and Ticku, 1983). Since the discovery of GABA transporters, it became clear that GABA could be accumulated in different cell types (neuronal and glial cells), suggesting the presence of different transport systems (Iversen and Kelly, 1975; Johnston, 1978; Hyden et al., 1986). To investigate GABA uptake inhibition, several analogues have been generated by systematic modification of the endogenous structure (See Figure 5A). Some of the GABA-analogues are used as neuronal markers (ACHAC, ß-alanine) (Iversen and Kelly, 1975; Krogsgaard-Larsen et al., 1981). This process led to the rational design of a rigid scaffold. An important step was the synthesis of 4,5,6,7-tetrahydroisoxazolo(5,4-c)pyridin-3-ol (THIP) and tetrahydroisoxazolo[4,5-c]pyridin-3-ol (THPO). The former compound has a high affinity for GABA receptor, and the latter is a potent inhibitor of the GATs but has no affinity for the GABA receptors (Schousboe et al., 1981). These compounds allowed for the first time to isolate the effects mediated by the receptor from those originating from transporter activity. The activity of various transporter systems determines the balance of uptake into glial vs. neuronal cells. The availability of exo-THPO, which showed selectivity for glia cell transporters, allowed to differentiate GABA uptake into glial over neuronal uptake (Schousboe et al., 1979; Falch et al., 1999). In further developments, the conformationally restricted GABA analogue THPO becomes the scaffold for designing potent and selective GAT1 (neuronal) inhibitors, whereas exo-THPO led the design of GAT2 (hBGT-1) inhibitors (Clausen et al., 2005).

The first available inhibitors nipecotic acid and guvacine, synthesized through bioisostere substitution of the isoxazole to carboxylic acid of the THPO scaffold, failed to cross the blood-brain barrier (BBB) (Schousboe et al., 2004; Schousboe et al., 2014; Schousboe et al., 2017). Both compounds are potent and selective *in vitro* inhibitors of GAT1, and share the common feature of being zwitterionic, which retains a similarity to the endogenic zwitterionic GABA, but also makes them poorly permeable through the BBB because of the simultaneous presence of a positive and a negative charge (Johnston et al., 1975; Johnston et al., 1976a). To increase brain penetration, the molecules were modified through esterification of the carboxylic acid, creating mono and diesters prodrugs (Frey et al., 1979; Croucher et al., 1983; Falch et al., 1987). The resulting molecules were effective on seizure modulations (in rats), but showed limited potency and non-specific side effects mediated by activity on muscarinic receptors, thereby limited their applicability (Frey et al., 1979; Falch et al., 1987). In an attempt to increase the lipophilicity of the active molecules (See Figure 5B), a dipheniylbut-3-en-1-yl (DPB) group was introduced on the secondary amino group of guvacine, (*R*)-nipecotic acid, and THPO: the diaromatic chain-substituent analogues resulted in the highly potent and orally active GABA uptake inhibitors SKF89976-A and SKF100330-A (Bolvig et al., 1983; White et al., 1983; Ali et al., 1985). Moreover, the inhibition kinetics associated with these compounds were different from the previously reported competitive inhibitors, because instead these molecules were not translocated as substrate, but rather acted as blockers (Johnston et al., 1976b; Larsson et al., 1988). This discovery started the development of several lipophilic analogues that eventually resulted in selective GAT1 vs. GAT2-3 and BGT-1 inhibitors (Dhar et al., 1994). Further developments of these hGAT1 selective compounds increased the BBB permeability and resulted *in vivo* anticonvulsive activity of CI-966(([1-[2-[bis-4-(trifluoromethyl)-phenyl]methoxy]ethyl]-1,2,5,6-tetrahydro-3-pyridinecarboxylic acid)), which entered phase 1 clinical trial, but the compound suffered from severe side effects such as myoclonus and catatonic-like states (Sedman et al., 1990; Taylor et al., 1990). Further developments were achieved by introducing an oxime in the compound NNC-711 (1-(2-(((diphenylmethylene)amino)oxy)ethyl)-l,2,4,6-tetrahydro-3-pyridinecarboxylic acid hydrochloride (Smith et al., 1995). Finally, tiagabine, an isoxazole bioisoster of the aromatic moiety, emerged as a potent and selective GABA uptake inhibitor and became the first approved drug for the treatment of epilepsy (Braestrup et al., 1990; Nielsen et al., 1991; Halonen et al., 1996; Meldrum, 1996; Kern and Wanner, 2015). Currently, the lipophilic analogs tiagabine (NO-328) and NNC‐711 (1-(2-(((diphenylmethylene)amino)oxy)ethyl)-1,2,5,6-tetrahydro-3- pyridinecarboxylic acid hydrochloride) are approved as the selective antiepileptic drugs, but the severe cognitive side effects (confusion, abnormal mood swings, dizziness, tremor, fatigue, nervousness) caused by the tiagabine limits their applicability and raises the need for alternative medication (Krogsgaard-Larsen et al., 1979; Krogsgaard-Larsen et al., 1981; Fariello and Ticku, 1983; Schousboe et al., 2004; Schousboe and Madsen, 2017).

In 2014, a series of N-substituted guvacine derivatives have been screened as novel GAT1 inhibitors, which lead to the identification of DDPM-2571, which is a di-chloro-phenyl derivative that showed a four-time higher potency (Kern and Wanner, 2015). Screening o5-substituted hydrazones of nipecotic acid resulted in the first allosteric modulator of GAT1 (Hauke et al., 2018). Selective pharmacology towards BGT-1 started when EF1502 was developed from N-Me-exo-THPO and tiagabine. It showed equal inhibition efficacy for GAT1 and BGT-1, but not for GAT2 and GAT3, whereas its S-enantiomer was the first BGT-1 selective inhibitor with moderate affinity. This S-enantiomer of EF1502 showed anticonvulsive effects *in-vivo* experiments (Smith et al., 2008). If combined with tiagabine, a synergistic effect could be observed, suggesting that BGT-1 has a functional role in the CNS and supporting the notion of the importance of BGT-1 as a target for anticonvulsant drugs (White et al., 2005; Smith et al., 2008). To further increase the selectivity for BGT-1, the lipophilic side chain of tiagabine was combined with different amino-acidic groups, which led to the conformationally restricted analogue RPC-425, selectivity towards BGT-1; in combination with tiagabine the effect is not synergistic but additive, indicating different pharmacological effect or apparent mechanism (Vogensen et al., 2013).

In a different approach aiming at gaining affinity and selectivity, the conformation of GABA was restricted with a cyclopropane ring (Roberts and Frankel, 1950) (Nakada et al., 2013) (See Figure 5C). It was observed that when the cyclopropyl and carboxyl group were on the same side (syn conformation), the compound (Udenfriend, 1950) has the highest biological activity, resulting in the currently most potent BGT-1 inhibitor (Kobayashi et al., 2014). Based on nipecotic acid as a template, the bioisosteric substitution of the amino group with guanidine resulted in the selective BGT-1 inhibitor 2-amino-1,4,5,6-tetrahydropyrimidine-5-carboxylic acid (ATPCA) (Al-Khawaja et al., 2018). Interestingly, the pyridine derivative (Awapara et al., 1950) decreased the potency, indicating the importance of maintaining the position of the guanidinium N^3^ and carboxylic acid, respectively, in an orientation that is similar to ß-alanine. In regards of non-amino acid scaffolds, substitution of the GABA carboxyl group with a hydroxyl group led to the synthesis of the BGT-1 selective compound 1-(3-(9H-carbazol-9-yl)-1-propyl)-4-(2-methoxyphenyl)-4-piperidinol (NNC 05-2090) that showed 10-fold selectivity towards BGT-1 and K_i_ = 1.4 ± 0.3 μm, thereby proving that the amino acid scaffold was not necessary to target and inhibit BGT-1 (Thomsen et al., 1997). Derived from the scaffold of NCC 05-2090, N-(1-benzyl-4-piperidinyl)-2,4-dichlorobenzamide (BPDBA) was then identified as the first non-competitive inhibitor of BGT-1, suggesting a possible allosteric mode of interaction (Kragholm et al., 2013; Kickinger et al., 2020). More recently, selectivity of inhibitors towards BGT-1 were explored by developing analogs of ATPCA and bicyclic N-methylated GABA, which showed increased selectivity for BGT-1 at the expense of affinity (Kickinger et al., 2020; Kickinger et al., 2021).

## 6 Computational studies in hGAT1

**FIGURE 6:**
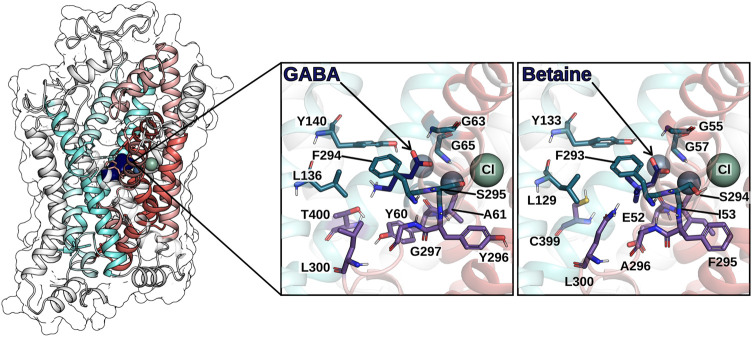
hGAT1 is shown in ribbon representation and in transparent surface rendering. The coloring scheme follows Figure 4: TM helices of the scaffold domain are shown in cyan colors, the TM helices of the bundle domain in red colors. GABA (in the S1) is shown by dark blue van der Waals rendering. The zoom-in panels highlight the residues on the surface of the S1 for the GABA bound GAT1 and the betaine bound BGT-1, respectively. Spheres indicate the sodium ions (grey-blue) and the chloride ion (grey-green).

In 2005, the first crystal structure of LeuT from *A. aeolicus* was resolved in an outward-facing occluded conformation, in which the substrate binding site S1 was occupied by the substrate (leucine) and shielded by the hydrophobic gate residues (V104, Y108, and F253) from the extracellular environment (Yamashita et al., 2005). The LeuT structures served as the template for the generation of GAT1 homology models, all owing for investigating the binding mode of GABA and analogues (Pallo et al., 2007; Skovstrup et al., 2010; Skovstrup et al., 2012; Zafar and Jabeen, 2018; Zafar et al., 2019; Latka et al., 2020). The endogenous ligand (GABA) was docked in the S1 and showed favorable h-bonding interaction with TM1 (Y60) and TM8 (S396)) (Pallo et al., 2007). The amino and carboxyl groups of GABA overlapped with the respective groups of leucine as observed in LeuT, whereby the carboxyl group of GABA completed the octahedral coordination of the sodium ion. A more extended conformation of GABA was proposed later (Wein and Wanner, 2010). In this pose, the amino group interacts with side chains of T400 and S396 on TM8, while interacting with the backbone of Y60 of S396. The hydroxyl group of Y140, the nitrogen of G65, and the backbone oxygen of F294 contributed a stabilizing network of hydrogen bonds, whereas T400 and G297 were proposed to be major contributors to GABA selectivity (Wein and Wanner, 2010). Skovstrup et al. (2010) described the S1 site in the occluded conformation as a pocket, in which the backbone of A61 and Y296 (I53 and F295 in BGT-1), the side chains of Y60, G297, L300, T400 build the lower floor, while the residues G63, G65, L136, Y140, F294, S295, S396 complete the S1. Interestingly, the latter group of residues is fully conserved within the GAT subfamily, whereas the floor of the pocket presents some difference that could be key in the transporter selectivity. The recent structure of GAT1 in the inward-facing conformation and the AlphaFold model in the outward-facing conformation confirmed the prediction for most residues (See Figure 6). In particular, Y60 of GAT1 (E52 in BGT-1) is proposed to contribute to substrate specificity, together with T400, as both residues differ within the GAT subfamily (Melamed and Kanner, 2004; Baglo et al., 2013).

A later docking study using a GAT1 homology model based on the X-ray crystal structure of the open-to-out conformation of the dDAT showed a GABA binding mode that agreed with the earlier studies, in which the carboxyl group completes the coordination of Na^+^ in Na1, while the amino group is mainly stabilized by interactions with Y140 and Zafar and collaborators reported that the presence of the co-transported Na^+^ and Cl^−^ ions increase binding strength, in particular Na^+^ bound to the Na1 site (Wang et al., 2015; Zafar et al., 2019).

Consistent with the binding pose of GABA, flexible docking of the short inhibitors (nipecotic acid, guvacine, 4-amino-isocrotonic, taurine, and 4-amino-2-hydroxubutanoic-acid) to the S1 binding site of GAT1 models derived from the substrate-bound outward-occluded conformation of LeuT showed the same polar contacts (Wein and Wanner, 2010). When comparing the binding of nipecotic acid with the binding pose of tiagabine, an additional stabilizing interaction formed with the backbone of F294 that adopts a different orientation in respect of the occluded conformation, suggesting that larger inhibitors act as blockers by stabilizing the outward-open conformation and preventing occlusion (Skovstrup et al., 2010).

Studies of the translocation pathway of substrates in GAT1 were performed by steered molecular dynamics simulation, in which a pulling force was applied on the substrate to bias it towards leaving the S1 towards both the extracellular and the intracellular side (Skovstrup et al., 2012). The study recapitulated the S2 in the extracellular vestibule in a similar position as first identified in LeuT (Shi et al., 2008). The charged residues K76, K278, D281, E283, D287 appear to attract and guide the substrate towards the S2: related mutants show a loss in substrate transport supporting the role of these residues in substrate association (Keshet et al., 1995; Rosenberg and Kanner, 2008). E101 was suggested to play a role in the translocation of substrate from the S1 to the intracellular side (Keshet et al., 1995). For the inhibitor nipecotic acid, a similar dissociation path as for GABA was observed, whereas tiagabine showed stronger hydrophobic interactions with TM1 and TM6, most extensively with residues between the S1 and S2 sites (Skovstrup et al., 2012). Docking studies of nipecotic acid to GAT1 showed poses that place the amino nitrogen towards the intracellular side forming a hydrogen bond with the F294 backbone (Pallo et al., 2007; Wein and Wanner, 2010). Docking of tiagabine also showed a similar ligand-amine to F294 interaction, but the large tiagabine could not be fitted into the too-small binding site of the occluded-state and its thiophene rings are positioned towards the extracellular side (Rosenberg and Kanner, 2008; Skovstrup et al., 2010). This size dependence suggested that the inhibitory kinetics should depend on the binding mode, as smaller inhibitors fit perfectly into the closed site S1 and their nitrogen forms a hydrogen bond with the oxygen atoms of Y60 located at the floor of the S1 (Wein et al., 2016). The binding mode of tiagabine was also investigated through experiment-guided docking and molecular dynamics simulation by Jurik et al. (2015). The orthosteric S1 binding site was described as a space divided into two hydrophobic cavities by S1-exposed residues: the sub-pockets are confined by the side chains of I143 and Y140 and by W68, F294, A358, whereby each sub-pocket accommodates an aromatic moiety of the inhibitor. This description of the binding site is in accordance with the results of ligand-based drug design studies, in which a polar atom in the linking region between the two aromatic moieties of inhibitors resulted in increased binding affinity (Knutsen et al., 1999).

Recently, the first cryo-EM structure of full-length wild-type hGAT1 in complex with tiagabine was resolved (Motiwala et al., 2022). The electron density of tiagabine in the inward-open conformation adopts an orientation that differs from the predicted conformations, as the aromatic moieties are oriented towards the bottom of the binding pocket and interact with Y60, L303 and L306, whereas the nipecotic acid moiety is located between the two sodium-binding sites Na1 and Na2.

## 7 Energetics of the transport cycle

### 7.1 Energy coupling/third binding site

The switching from the inward-facing to the outward-facing state requires transporter isomerization in the absence of the substrate and the co-transported ions, which seems to be the rate-limiting step and accordingly defines the overall transport velocity. The transport cycle of GATs is energized by the transmembrane gradient of Na^+^ and utilized for the co-transport of the substrate, thus accelerating the transition of the outward-facing state to the inward-facing state in the presence of substrate and ions. This allows cellular accumulation of substrate by GATs as long as the transmembrane electrochemical gradient of sodium is present. The stoichiometry for the GATs remains disputed between 2 or 3 sodium ions per transported GABA as described in Section 4.8 (Radian and Kanner, 1983; Keynan and Kanner, 1988; Mager et al., 1996; Matskevitch et al., 1999; Matthews et al., 2009; Omoto et al., 2012; Willford et al., 2015; Eskandari et al., 2017). According to the Nernst equation, the accumulating power depends on the stoichiometry of transport, which enters as an exponent to the equation. A stoichiometry of 3 Na^+^ per GABA vs. 2 Na^+^ per GABA leads to an energy difference in the concentrative power of ∼14 kJ/mol at physiological concentrations. Similar to the GABA transporters, also the glycine transporters (GlyT1 and GlyT2) have different stoichiometries: GlyT1 (expressed in glia cells) has a stoichiometry of 2 Na^+^: 1 Cl^-^: 1 glycine, while GlyT2 (located on pre-synaptic neurons) shows a stoichiometry of 3 Na^+^: 1 Cl^-^: 1 glycine for GlyT2. The different stoichiometry of GAT1 and BGT-1 could implicate difference of roles they play in CNS, especially in terms of substrate accumulation.

### 7.2 Proposed kinetic models of transport

A useful tool in the description of the secondary active transport is the kinetic model, a scheme that tries to allocate individual reaction steps within the transport cycle, and by estimating the rates for each step it can predict the experimental results (Burtscher et al., 2019). For secondary active transporters, the most generally accepted hypothesis explaining the stoichiometric transport of substrates is the “alternating access” model, stating that substrates can bind at each time from one side of the membrane only, and accessibility alternates depending on the conformational state of the transporter (Jardetzky, 1966).

**FIGURE 7:**
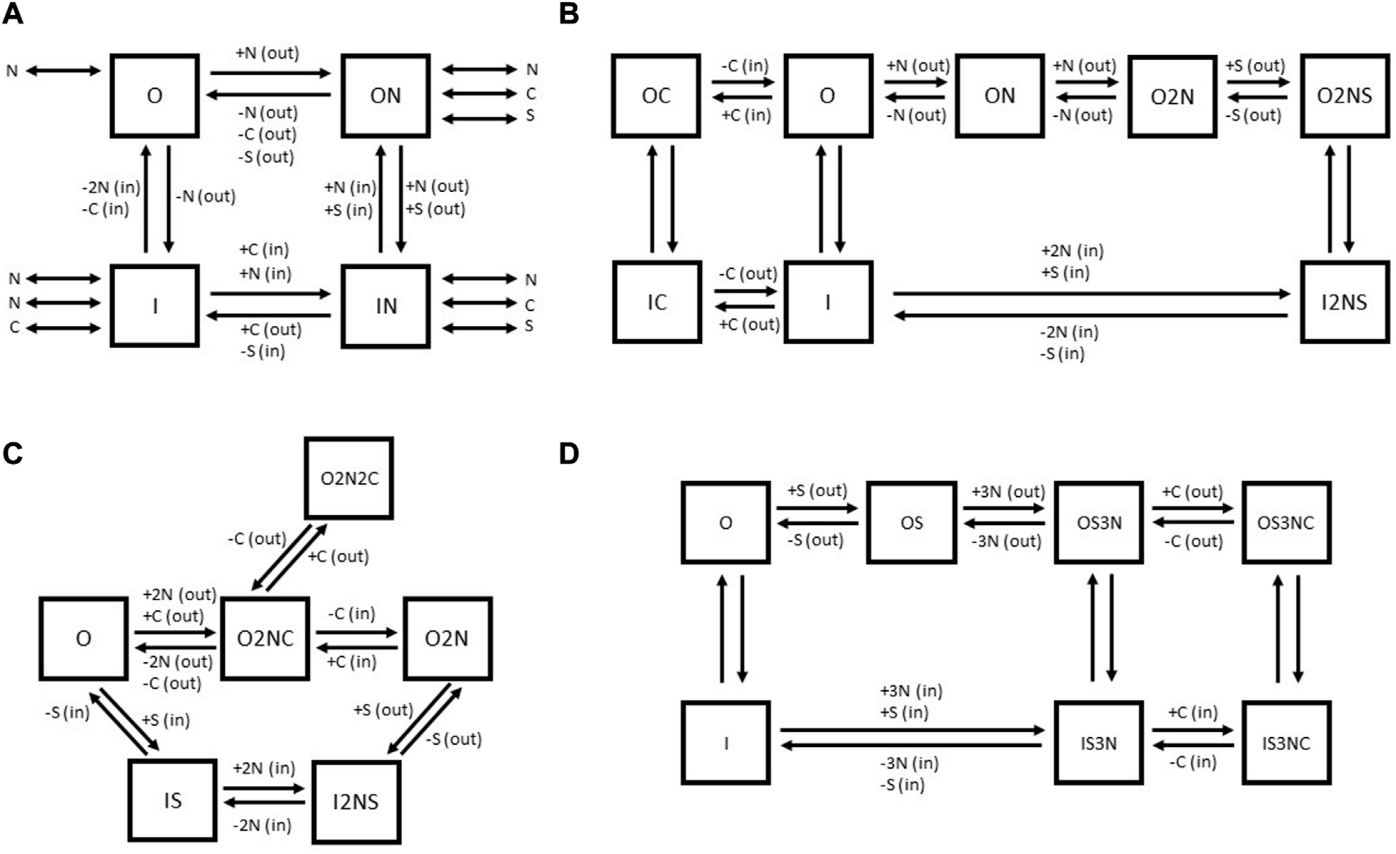
Comparison of published kinetic schemes for GAT1 **(A–C)** and BGT-1 **(D)**. The outward-facing states of transporters are indicated by the letter “O,” while the inward-facing states are indicated by the letter “I.” “N” indicates Na^+^, “C” indicates Cl^−^ and “S” indicates the substrate GABA (for both GAT1 and BGT-1) or betaine (for BGT-1). In parenthesis, (out) or (in) indicates the extracellular or intracellular localization of the respective substrate. For the schemes **(B–D)**, the (+) and (−) signs alongside the substrates near the individual reaction arrows indicate the binding (+) or unbinding (−) of the respective substrate. The kinetic scheme **(A)** is adapted for GAT1 from Hilgemann and collaborators (Hilgemann and Lu, 1999), **(B)** is based on the kinetic scheme for GAT1 proposed by Bicho and collaborators (Bicho and Grewer, 2005). The kinetic scheme **(C)** for GAT1 was originally proposed by Cherubino et al. (2012). **(D)** The kinetic scheme for BGT-1 was proposed by Matskevitch et al. (1999).

For GAT1, a certain number of models have been proposed (See Figure 7). Based on the impossibility to detect leak currents in *X. laevis* oocytes, an early model by Hilgemann and Lu (1999) proposed a four-state kinetic model (See Figure 7A). This model assumes that the empty carrier facing the cytoplasmic side and the outward-facing carrier with one bound Na^+^ ion resemble the stable states of the cycle, whereas the empty carrier facing the extracellular side and the inward-facing carrier with one Na^+^ bound are assumed as “metastable” states capable of accumulation during the reverse transport. The (+) or (−) signs alongside the substrates near the individual reaction arrows indicate whether the respective substrate must bind to the carrier for the reaction to occur (+) or if it must be released from the carrier for the reaction to proceed (−) (See Figure 7A). The substrates indicated by the double-headed arrows are considered in a dissociation equilibrium with the carrier. It is important to note that each double-headed arrow represents an intermediate state not directly shown in the model. The authors suggested ordered binding, while the different conformational transitions were expected to conclude only when a particular combination of ions is bound. For further details, we refer to the original publication (Hilgemann and Lu, 1999). The authors attributed a high electrogenicity to the conformational change because the alternating exposure of the Na^+^ binding site to either side of the membrane implies that charges associated with the Na^+^ binding site cross the electric field of the membrane during the conformational isomerization. This isomerization reaction depends on the extracellular Na^+^ concentration; Na^+^ binding to the empty carrier in the outward-facing conformation shifts the equilibrium within the two empty carrier states, i.e., reduces the amount of inward-facing empty carriers that undergo the conformational transition. The negative charge moved across the membrane during this transition was predicted to account for the majority of the charge transferred during the whole transport cycle, because it is not neutralized by bound Na^+^ ions as in the substrate-bound carrier. This model was able to predict accurately the data from *X. laevis* oocytes but failed to explain uncoupled currents observed by others (Fesce et al., 2002; Krause and Schwarz, 2005).

Bicho and Grewer (2005) tried to refine the kinetic model of GABA transport and to include the role of Cl^−^ by exploiting experimental information coming from rapid substrate jumps (generated by pulsed-laser photolytic release of caged GABA) in combination with patch clamp measurements. The major electrogenicity in this model was associated with the reaction “O” to “ON” (the binding of the first Na^+^ ion to the empty, outward-facing carrier) (See Figure 7B). The authors were able to resolve two fast reactions, which are weakly electrogenic and associated with conformational rearrangements of GAT1 upon GABA binding, suggesting that the change in exposure of the S1 from extracellular to intracellular moves a small amount of charge across the membrane electric field. This kinetic model proposes that the Na^+^ ions bind sequentially and thereby enhance the affinity of GAT1 for GABA. Then GABA binds in a non-electrogenic reaction, a step followed by GAT1 orienting to the inward-facing state through a weakly electrogenic passage and successively by the release of the substrates. At this point, external Cl^−^ is predicted to bind to the inward-facing transporter, allowing it to switch to the outward-facing conformation. In the absence of external Cl^−^, only a slow (rate-limiting) reorientation of the empty carrier can take place. In this model Cl^−^ transport is considered independent from GABA translocation. The effect of Cl^−^ would be to accelerate GAT1 reorientation from the inward-facing to the outward-facing state, thereby enhancing apparent affinity of GAT1 for Na^+^ and GABA and accelerating the overall transport rate (See Figure 7B). The first model after solving the structure of the SLC6 transporter family aimed to propose a kinetic scheme that accounts for interactions of intracellular substrates and included experimental data observed with different intracellular conditions on pre-steady state currents and reverse transport reaction employing TEVC on *X. laevis* oocytes (See Figure 7C) (Cherubino et al., 2012). Cherubino et al. (2012) noticed that an increase in cytoplasmic Cl^−^ can accelerate the rate constant of outward Na^+^-induced pre-steady state currents, while the same rate constant is decreased by increasing extracellular Cl^−^. To include this phenomenon in the kinetic model, the authors proposed the existence of a state outside the transport cycle in which GAT1 is sequestered by a second external Cl^−^ anion after the inward movement of charges. This would decrease the outward rate of the charge movement and the reverse transport current. In this scheme, the electrogenic step is a single step in which all the co-substrates bind to the transporter in the outward-facing conformation (“O” to “O2NC”). The out-of-the-cycle state “O2N2C” that binds two Cl^−^ is necessary to explain the decrease of outward Na^+^-induced transient, while the step “O2NC” to “O2N” is required to explain the opposite effect of intracellular Cl^−^ on the outward Na^+^-induced transient.

For BGT-1, Matskevitch et al. (1999) draw three important conclusions from their data collection: (Awapara et al., 1950): the maximum velocity of GABA uptake decreases with decreasing external Na^+^ and Cl^−^, (Roberts and Frankel, 1950), the maximum velocity can still be reached at the sub-saturating GABA concentrations, (Udenfriend, 1950), there is still significant transport in the absence of Cl^−^. These considerations would be consistent with a model in which GABA or betaine binds first to a 2 Na^+^ bound BGT-1, followed by the binding of third Na^+^. At this point, the transporter can orient in the inward-facing state even in absence of Cl^−^, through a slow reaction. The presence of Cl^−^ consents to the binding at a faster rate, augmenting the maximum velocity of the transporter (See Figure 7D).

## 8 Conclusion

Since its discovery, GAT1 has been extensively studied and targeted for treating neuropathological disorders, while BGT-1 remains understudied. In contrast to GAT1, BGT-1 is not localized in GABAergic synapses and its GABA transport efficiency is lower than GAT1, hence its function in the brain may be mainly related to its ability to transport betaine. While the potent inhibitors of GAT1 are used to treat epileptic seizures, the *in vivo* studies demonstrating the ability of BGT-1 to impact the severity of epileptic seizures, raised a possibility of functional similarities between GAT1 and BGT-1. The first class of GABA transporter inhibitors (guvacine and nipecotic acid) are also transporter substrates, while tiagabine acts as an inhibitor, a behavior that was predicted by computational studies and supported by experimental data: the drug stabilizes the outward-open conformation by stretching from the S1 to the S2. The first cryo-EM structure of tiagabine-bound GAT1 suggests a different mode of interaction, as tiagabine was found to bind the inward-facing state of GAT1. Resolving this discrepancy is important for understanding transporter inhibition and for further improving currently available medication targeting the GABA transporters. The recent developments of BGT-1 selective inhibitors based on the GAT1 inhibitors (N-Me-exo-THPO and tiagabine) show that using GAT1 as a research model could effectively further the study of BGT-1.

A deeper understanding of the ion dependence and their specific role in the transport reaction in GAT1 and BGT-1 may prove important in better defining the interaction of inhibitors and help designing new drugs. Both proteins are Na^+^ dependent, and substituting Na^+^ with other cations (e.g., K^+^) either blocks transport or activates a leaking state of the carrier (e.g., Li^+^). The role of Cl^−^ remains unclear, but undoubtedly Cl^−^ is important for achieving maximal transport functionality. It is postulated that Cl^−^ could have a compensating effect on the charges carried by the Na^+^ ions, but such a role could also be fulfilled by a generic negative charge. Evidence for this resides in the fact that some transport functions remain in absence of Cl^−^ at highly negative membrane potentials, and more specifically in the GAT1 mutant S331E, which can transport GABA in the absence of Cl^−^. In this GAT1 variant, the serine residue that is part of the Cl^−^ binding site has been substituted with glutamate, thereby effectively placing a negative charge into the space which Cl^−^ would occupy. The reason for the importance of Cl^−^ may lie in the protein structure as a significant number of residues involved in Cl^−^ binding are also involved in Na^+^ binding.

Another source of debate is the stoichiometry of transport. For BGT-1 a stoichiometry of 2 or 3 (Na^+^): 1 or 2 (Cl^−^): 1 (GABA/betaine) has been proposed, while for GAT1, it is unclear if 2 or 3 Na^+^ ions are co-transported with one GABA molecule and some authors propose a Cl^−^/Cl^−^ exchange mechanism. The order of ion and GABA binding remains unresolved from kinetic modelling studies and models also suggest that there could be a difference between GAT1 and BGT-1. The potential difference between GAT1 and BGT-1 is intriguing and could be exploited to better understand the origin of transporter selectivity. Therefore, there is a big need for more basic knowledge on BGT-1 and for expanding the existing knowledge on GAT1 to clarify these uncertainties to better understand their fundamental roles and to improve medication for patient treatment that suffers from transporter-related pathologies.
